# Cost-analysis of Implementing Robot-assisted Versus Open Pancreatoduodenectomy

**DOI:** 10.1097/SLA.0000000000006665

**Published:** 2025-02-10

**Authors:** Julia E. Menso, Michiel F.G. Francken, Nine de Graaf, Alessandro M. Bonomi, Riccardo Guastella, Dylan Balaban, Mahsoem Ali, Jaap Bonjer, Susan van Dieren, Marcel G.W. Dijkgraaf, Jony van Hilst, Gabriela Pilz da Cunha, Joris Erdmann, Geert Kazemier, Rutger-Jan Swijnenburg, Barbara M. Zonderhuis, Freek Daams, Sebastiaan Festen, Olivier R. Busch, Marc G. Besselink

**Affiliations:** * Department of Surgery, Amsterdam UMC, University of Amsterdam, Cancer Center Amsterdam, Amsterdam, The Netherlands; † Department of General Surgery and Biomedical and Clinical Sciences, ASST Fatebenefratelli Sacco, University of Milan, Milan, Italy; ‡ Department of Surgical, Oncological and Gastroenterological Sciences, General Surgery 2 Hepato-Pancreato-Biliary Surgery and Liver Transplantation, Padua University Hospital, Padua, Italy; § Department of Surgery, Amsterdam UMC, Vrije Universiteit Amsterdam, Cancer Center Amsterdam, Amsterdam, The Netherlands; ‖ Department of Epidemiology and Data Science, Amsterdam UMC, University of Amsterdam, Amsterdam, The Netherlands; ¶ Department of Surgery, OLVG, Amsterdam, The Netherlands

**Keywords:** cost-analysis, economic evaluation, minimally invasive pancreatic surgery, robot-assisted pancreatoduodenectomy

## Abstract

**Objective:**

To perform a cost analysis during the implementation of robot-assisted pancreatoduodenectomy (RPD) in a high-volume center.

**Background:**

Many high-volume centers are implementing RPD as an alternative to open pancreatoduodenectomy (OPD) but the persevering concerns about increased costs of RPD versus OPD have not been addressed by large studies.

**Methods:**

Post hoc cost analysis of consecutive RPD versus OPD in a singly high-volume center (January 2015–June 2024). The eligibility criteria for RPD (ie, no vascular contact, no chronic and necrotizing pancreatitis, and body mass index <35 kg/m^2^) were used to select patients undergoing OPD to minimize selection bias. Primary outcomes were total hospital costs and total hospital stay. Sensitivity analyses excluded the first half of the RPD experience.

**Results:**

Overall, 588 patients undergoing pancreatoduodenectomy were included (214 RPDs, 374 OPDs). Total hospital stay was shorter after RPD [10 days (6 to 17) vs 12 (8 to 21), *P* = 0.001]. Mean total hospital costs were €4804 higher for RPD (€27,307 vs €22,503, *P* = 0.010). Outcomes improved in the second half of the RPD experience (n = 107): total hospital stay [12 days (7 to 23) to 9 (6 to 15), *P* < 0.001], pancreatic fistula (48.6% to 31.8%, *P* = 0.012), delayed gastric emptying (33.6% to 15.0%, *P* = 0.001), and operative time [373 minutes (341 to 411) to 310 (272 to 352), *P* <0.001]. Consequently, costs per RPD procedure decreased by €11,058 (€32,836 to €21,778, *P* = 0.001). Costs in the second half of the OPD experience remained stable (€24,025 to €21,013, *P* = 0.210). Hence, total hospital costs for RPD and OPD became comparable in the second half (€21,778 vs €21,013, *P* = 0.644).

**Conclusions:**

Implementing RPD is associated with considerable additional costs compared with OPD. With growing experience, both the outcomes and cost-efficiency of RPD improve, with costs decreasing by approximately a third, leading to similar costs as OPD. Large-scale randomized trials will have to confirm these findings.

Pancreatoduodenectomy is a complex surgical procedure associated with high postoperative morbidity rates, frequent reinterventions, and prolonged hospitalization resulting in a high economic burden to health care systems.^1^ In selected patients, minimally invasive pancreatoduodenectomy is increasingly used as an alternative to open pancreatoduodenectomy (OPD) to enhance postoperative recovery and improve outcomes.^2,3^ In the Netherlands, the LEOPARD-2 randomized trial resulted in a nationwide shift from laparoscopic to robot-assisted pancreatoduodenectomy (RPD).^4^ Subsequently, a nationwide training program facilitated the safe implementation of RPD with currently 25% of all pancreatoduodenectomies being performed as RPD.^5^ Retrospective series have demonstrated promising clinical outcomes, such as reduced intraoperative blood loss and shorter hospital stay after RPD as compared with OPD.^6–8^ However, implementation of RPD faces scrutiny because of the high costs of the robotic platform and robotic instruments as compared with OPD.^6,8^

In many centers, the debate continues on whether the benefits of RPD outweigh the higher operative costs and longer operating times. However, these findings were reported in small cohorts of patients.^6,9–11^ With increased surgical experience, operating time will reduce, and clinical outcomes will improve,^5^ resulting in lower hospital costs of RPD. This hypothesis requires a detailed evaluation in large RPD and OPD cohorts from high-volume centers that have surpassed the learning curve. However, such studies are currently lacking.

Therefore, this study aimed to assess the hospital costs and clinical outcomes for RPD versus OPD in a high-volume tertiary referral center.

## METHODS

### Study Design

This is a post hoc study, including patients undergoing RPD and OPD, in Amsterdam UMC, The Netherlands. Ethical approval for the study was granted by the Medical Ethics Review Committee of the Amsterdam UMC. This manuscript was written in accordance with the CHEERS 2022 Statement.^12^ The CHEERS 2022 checklist is provided in Supplemental File 1 (Supplemental Digital Content 1, http://links.lww.com/SLA/F407).

### Patient Selection

Adult patients (≥18 years of age) who underwent RPD and OPD between January 2014 and December 2023 (for OPD) and June 2024 (for RPD) were included. Indication for surgery and choice of approach was decided upon in a multidisciplinary meeting including surgeons, oncologists, gastroenterologists, radiologists, and pathologists. Patient selection criteria for RPD remained similar during the entire study period: no vascular contact, no history of chronic and necrotizing pancreatitis, body mass index (BMI) <35 kg/m^2^, no pregnancy, and no secondary tumor requiring resection during the same procedure. All consecutive patients after RPD, from the first procedure onwards, were included. To minimize treatment allocation bias, all patients who underwent OPD were retrospectively screened and only included if they met the selection criteria for RPD.

### Data Collection

Data of all pancreatoduodenectomies performed at the Amsterdam UMC were extracted from the prospectively maintained Dutch Pancreatic Cancer Audit, the national mandatory audit registering all pancreatic procedures in the Netherlands since January 2014. Additional data were retrospectively obtained from electronic patient records. A bottom-up economic evaluation was conducted from a hospital perspective. All cost categories relevant to the surgical procedure and the postoperative course were included. The follow-up period included primary admission and readmission up to 30 days postoperatively. If the primary admission exceeded 30 days, the total duration of the primary admission was used.

Baseline characteristics were age, sex, BMI, American Society of Anesthesiologists score, preoperative diagnosis, tumor location, tumor diameter, and neoadjuvant therapy. Intraoperative variables were time in the operation room (OR; total time between patient arrival and exit from the OR), surgical time (time from initial incision to wound closure), anesthesia time (time before and after surgical time), blood loss, pancreatic duct size, pancreatic texture, venous and arterial resection, and length of planned post-anesthesia care unit (PACU) stay. Postoperative variables included procedure-specific complications,^13–17^ wound infection (according to the Centers for Disease Control classification^18^), organ failure, mortality, and health care use [eg, length of primary stay, total length of stay (including readmission), length of unplanned medium care (MC)/intensive care (IC) unit admission, postoperative imaging, radiologic, endoscopic, and angiographic reinterventions, reoperation, blood transfusion, emergency room (ER) visits, and unplanned outpatient clinic visits].

### Local Standard of Care

All patients underwent abdominal computed tomography and some also magnetic resonance imaging before surgery. Robot-assisted pancreatoduodenectomy (RPD) was implemented in 2018. All RPD procedures were performed by a standardized approach^19^ using the Da Vinci Xi Robotic Surgical System (Intuitive Surgical, Inc.). RPD was performed by a team of 2 surgeons (M.G.B. with O.R.B., S.F., or F.D.), who received formal training for robotic pancreatic surgery. Open pancreatoduodenectomy was performed by one of 6 pancreatic surgeons (O.R.B., F.D., G.K., J.E., B.M.Z., and M.G.B.) with 1 or 2 senior surgical residents. Postoperatively, patients were admitted to PACU, typically for 6 hours. Postoperative management was performed according to the Enhanced Recovery After Surgery (ERAS) guidelines and with similar protocols for both RPD and OPD.^20^

### Primary Outcomes

The primary outcome was total hospital cost (in-hospital/30 days postoperatively) and total length of hospital stay (LOS; ie, days of primary admission and days of readmission within 30 days).

### Secondary Outcomes

Secondary clinical outcomes (in-hospital/30 days postoperatively) were postoperative pancreatic fistula (POPF) grade B/C [International Study Group for Pancreatic Surgery (ISGPS) 2016 definition],^13^ postpancreatectomy hemorrhage (PPH) grade B/C (ISGPS definition),^14^ delayed gastric emptying (DGE) grade B/C (ISGPS definition),^15^ bile leak grade B/C [International Study Group of Liver Surgery definition],^16^ chyle leak grade B/C (ISGPS definition),^17^ mortality, MC/IC admission, reintervention, reoperation, and readmission. Secondary economic outcomes were intraoperative and postoperative costs.

### Costs

Costs were calculated for all patients individually by multiplying resource use with the corresponding unit costs of the respective cost driver (bottom-up). All costs were indexed to 2023 based on the consumer price indices reported by Statistics Netherlands (CBS).^21^ No discounting was applied.

Preoperative costs were not factored into the analysis as preoperative care was not expected to differ between the treatment groups. Intraoperative costs were calculated by combining fixed costs and additional costs. Fixed costs were procedure-specific costs, including a standard procedure-specific set of instruments and materials (RPD or OPD), and robot purchase costs in case of RPD. The set was based on the standard institutional content and included single-use disposable materials. Materials making a minor contribution to the costs (eg, gloves, gowns, and sutures) were disregarded in the cost analysis. The costs of instruments were calculated per procedure. Additional costs for an open set of materials were calculated in case of conversion during RPD. Purchase and maintenance costs of the robotic platform were factored into costs per procedure by equally dividing our hospital’s lease costs (2023) over the total of performed robot-assisted procedures in the hospital (€2150 VAT included). The current (2024) average manufacturer’s price (AMP) of Intuitive Surgical (Intuitive Surgical, Inc.) was set at €1850 per procedure. Additional intraoperative costs included costs for use of the OR per minute, equipment, personnel, and overhead. Additional intraoperative costs in the OR were sourced from a study that previously calculated OR prices from costs in 5 Dutch hospitals in a bottom-up assessment.^22^ Personnel wages included OR staff, 1 surgeon for OPD, and 2 surgeons for RPD. Postoperative costs were any costs made following exit from the OR until 30 days postoperatively or until discharge, if primary admission exceeded 30 days (including costs of hospital stay, MC/IC admission, diagnostic imaging, reintervention, reoperation, and readmission). A fixed cost was used for reoperation (different costs for relaparotomy and relaparoscopy), as standardly used by our center. Unit costs with references for each cost component are displayed in Supplemental File 2 (Supplemental Digital Content 1, http://links.lww.com/SLA/F407).^23^

### Statistical Analysis

Statistical analyses were performed with SPSS software version 28 for Windows (IBM SPSS Statistics). The primary analysis compared RPD versus OPD patients in the total cohort. As the RPD learning curve may have a considerable impact on the postoperative outcome,^5^ 2 additional analyses were performed: (1) comparing the first versus the second half of the RPD and OPD experience (50/50 method), and (2) comparing the second half of RPD patients versus the second half of OPD patients (to limit time bias). Sensitivity analyses encompassed: (1) excluding robot purchase/maintenance costs, (2) using the recently proposed AMP finance structure, (3) excluding the first 84 RPD procedures (according to the mastery learning curve^5^), and (4) excluding patients with cancer in the pancreatic head (ie, pancreatic ductal adenocarcinoma and distal cholangiocarcinoma) and chronic pancreatitis in the pancreatic head (ie, only high fistula risk remaining). Categorical data were analyzed using the χ^2^ test and presented as frequencies and percentages, as deemed appropriate. The distribution of continuous data was assessed with histograms. Normally distributed continuous data were analyzed with the *t* test and presented as means with SDs. Non-normally distributed continuous data were analyzed with the Mann-Whitney *U* test and expressed as medians with interquartile ranges. Missing values were single-imputed with 10 iterations, based on: age, BMI, tumor diameter, preoperative diagnosis, surgical approach, American Society of Anesthesiologists score, non-missing surgical time, non-missing anesthesia time, and non-missing total OR time. Differences in costs between groups were analyzed with the *t* test using nonparametric, bias-corrected, and accelerated bootstrapping by drawing 5000 samples and reported as means with bias-corrected and accelerated (BCa) 95% confidence intervals (CI). *P* values below 0.05 were considered statistically significant.

## RESULTS

### Clinical Outcome

In total, 588 patients undergoing pancreatoduodenectomy were included: 214 patients undergoing RPD between February 2019 and June 2024 and 374 patients undergoing OPD between January 2014 and December 2023. When applying the RPD eligibility criteria to patients undergoing OPD, 189 of 563 patients were excluded leaving 374 patients after OPD.

The total LOS (primary clinical outcome) was shorter after RPD compared with OPD [10 days (6 to 17) vs 12 (8 to 21), *P* = 0.001]. Length of primary stay was also shorter after RPD [9 days (6 to 15) vs 11 (7 to 19), *P* < 0.001] and more patients were discharged ≤6 days after RPD as compared with OPD [54 (25.5%) vs 46 (12.3%), *P* < 0.001]. The prevalence of cancer in the pancreatic head was lower in the RPD group compared with OPD [49 (22.9%) vs 182 (48.7%), *P* < 0.001]. No patient underwent RPD or OPD for chronic pancreatitis in the pancreatic head. Conversion occurred in 3.3% of RPD patients. Operative time was longer in the RPD group compared with OPD [346 minutes (301 to 388) vs 255 (219 to 315), *P* < 0.001], whereas intraoperative blood loss was lower with RPD [200 mL (100 to 300) vs 350 (219 to 600), *P* < 0.001]. The rate of POPF grade B/C was higher following RPD compared with OPD [86 (40.2%) vs 105 (28.5%), *P*=0.004], whereas sensitivity analysis excluding patients with cancer in the pancreatic head showed no significant difference in POPF rate (43.6% vs 34.9%, *P* = 0.093). Fewer chyle leak grade B/C [0 (0%) vs 16 (4.3%), *P* = 0.002] and fewer wound infections [43 (20.2%) vs 149 (39.9%), *P* < 0.001] were seen after RPD compared with OPD. Readmission rate was higher after RPD compared with OPD [49 (22.9%) vs 54 (14.4%), *P* = 0.009], and more patients visited the ER [45 (21.0%) vs 45 (12.0%), *P* = 0.004]. No difference in in-hospital/30-day mortality was found between RPD and OPD [4 (1.9%) vs 12 (3.2%), *P* = 0.337]. Details on the baseline characteristics of the total cohort are presented in Table 1 and the intraoperative and postoperative outcomes in Table 2.

**TABLE 1:** Baseline Characteristics of RPD Versus OPD (Total Cohort)

Baseline characteristics	Total cohort (n = 588)	RPD (n = 214)	OPD (n = 374)	*P* ^*^
Age (yr)	69 (61 to 74)	70 (61 to 74)	69 (61 to 75)	0.504
Sex (F)	237 (40.3)	86 (40.2)	151 (40.4)	0.964
BMI (kg/m^2^)	24.9 (22.5 to 27.8)	25.0 (22.4 to 28.0)	24.7 (22.5 to 27.7)	0.464
ASA score: 3 or higher	154 (26.2)	57 (26.6)	97 (25.9)	0.853
Cancer in the pancreatic head^†^	231 (39.3)	49 (22.9)	182 (48.7)	**<0.001**
Tumor diameter (mm)^‡^	24 (17 to 33)	23 (16 to 33)	24 (17 to 34)	0.939
Neoadjuvant therapy				0.669
Chemoradiation	10 (1.7)	4 (1.9)	6 (1.6)	NA
Chemotherapy	16 (2.7)	8 (3.7)	8 (2.1)	NA
Radiotherapy	2 (0.3)	1 (0.5)	1 (0.3)	NA

Bold values are the total costs or showed a stastically significant difference between the groups.

Values are presented in medians with interquartile ranges and frequencies with percentages (%).

* *P* value was generated based on difference between RPD versus OPD cohort.

† Here defined as both pancreatic ductal adenocarcinoma and distal cholangiocarcinoma.

‡ Reported in 454/588 patients.

NA indicates not applicable.

**TABLE 2:** Outcome and Costs of RPD Versus OPD (Total Cohort)

	RPD (n = 214)	OPD (n = 374)	*P*	Mean difference (Costs)
Intraoperative outcomes				
Conversion	7 (3.3)	NA	NA	NA
Operative time (min)	346 (301 to 388)	255 (219 to 315)	**<0.001**	NA
Anesthesia time (min)	66 (60 to 75)	65 (54 to 77)	0.163	NA
Blood loss (mL)	200 (100 to 300)	350 (219 to 600)	**<0.001**	NA
Vein resection	7 (3.3)	23 (6.2)	0.124	NA
Arterial resection	0	3 (0.8)	0.188	NA
Small MPD	129 (60.8)	208 (65.6)	0.264	NA
Soft pancreatic texture	134 (65.0)	203 (61.9)	0.462	NA
PACU stay (h)	16 (6 to 19)	16 (7 to 20)	0.770	NA
Postoperative complications				
POPF (grade B/C)	86 (40.2)	105 (28.5)	**0.004**	NA
DGE (grade B/C)	52 (24.3)	103 (27.5)	0.391	NA
Bile leak (grade B/C)	13 (6.1)	31 (8.3)	0.326	NA
PPH (grade B/C)	22 (10.3)	39 (10.4)	0.955	NA
Chyle leak (grade B/C)	0	16 (4.3)	**0.002**	NA
Wound infection (CDC)			**<0.001**	NA
Superficial	13 (6.1)	38 (10.2)	NA	NA
Deep	1 (0.5)	3 (0.8)	NA	NA
Intra-abdominal	29 (13.6)	108 (28.9)	NA	NA
Organ failure	11 (5.1)	26 (7.0)	0.376	NA
Postoperative health care use				
Length of primary stay (d)	9 (6 to 15)	11 (7 to 19)	**<0.001**	NA
Total length of stay (d)*	10 (6 to 17)	12 (8 to 21)	**0.001**	NA
≤6 d	54 (25.2)	46 (12.3)	**<0.001**	NA
Diagnostic imaging	144 (67.3)	263 (70.3)	0.444	NA
Endoscopy	85 (39.7)	134 (35.8)	0.348	NA
Radiologic intervention	99 (46.3)	148 (39.6)	0.114	NA
Reoperation	18 (8.4)	32 (8.6)	0.952	NA
MC/IC admission	17 (7.9)	44 (11.8)	0.144	NA
Blood transfusion	27 (12.6)	31 (8.4)	0.103	NA
Readmission	49 (22.9)	54 (14.4)	**0.009**	NA
Outpatient hospital care				NA
ER visit	45 (21.0)	45 (12.0)	**0.004**	NA
Outpatient clinic	0	3 (0.8)	0.189	NA
Mortality (in-hospital/30 d)	4 (1.9)	12 (3.2)	0.337	NA
Costs (EUR)				
Total intraoperative costs	**12,245 (12,086 to 12,414)**	**5616 (5534 to 5700)**	**<0.001** **†**	**−6629 (−6816 to −6449)**
Fixed	6440^	1947^	**<0.001** **†**	**−4493** **‡**
Additional	5805 (5646 to 5974)	3669 (3588 to 3754)	**<0.001** **†**	**−2136 (−2323 to −1956)**
Total postoperative costs	**15,062 (12,602 to 17,947)**	**16,902 (14,730 to 19,360)**	**0.308** **†**	**1840 (−1601 to 5361)**
Admission	11,616 (9764 to 13,694)	13,990 (12,265 to 15,901)	0.106 **†**	2374 (−511 to 5227)
Outpatient hospital care	69 (50 to 89)	35 (25 to 45)	**0.003** **†**	**−34 (−56 to −12)**
Diagnostic imaging	623 (512 to 743)	566 (495 to 640)	0.439 **†**	−57 (−202 to 84)
Endoscopy	624 (494 to 765)	549 (457 to 646)	0.389 **†**	−75 (−247 to 88)
Radiologic interventions	1389 (1073 to 1741)	1146 (952 to 1354)	0.249 **†**	−243 (−654 to 156)
Surgical reinterventions	538 (312 to 774)	527 (358 to 700)	0.955 **†**	−10 (−329 to 299)
Other	204 (119 to 302)	113 (65 to 167)	0.110 **†**	−92 (−206 to 16)
Total costs (intraoperative and postoperative)	**27,307 (24,783 to 30,247)**	**22,503 (20,328 to 24,944)**	**0.010** **†**	**−4804 (−8277 to −1264)**
Excl robot purchase costs	25,157 (22,633 to 28,097)	22,503 (20,328 to 24,944)	0.146 **†**	−2654 (−6127 to 886)

Bold values are the total costs or showed a stastically significant difference between the groups.

Values are presented in medians with interquartile ranges and frequencies with percentages (%). Costs are reported as means with mean differences and their 95% BCa CIs.

* Total length of stay includes readmission(s).

† *P* value was generated based on mean difference between costs.

‡ No CIs could be generated.

CDC indicates Centers for Disease Control; EUR, euro; MPD, main pancreatic duct; NA, not applicable.

### Hospital Costs

Total hospital procedural costs were €4804 higher for RPD (€27,307 vs €22,503, *P* = 0.010) as compared with OPD. Intraoperative costs (including €2150 per procedure for purchase and maintenance costs of the robotic platform) were higher for RPD than OPD (€12,245 vs €5616, *P* < 0.001), whereas postoperative costs did not differ (€15,062 vs €16,902, *P* = 0.308; Table 2).

When excluding robot purchase costs, total hospital costs did not differ between RPD and OPD (€25,157 vs €22,503, *P* = 0.146). The 2024 AMP finance structure (€1850 per procedure; replacing €2150) did not have an impact on total hospital costs of RPD versus OPD [€27,007 (95% BCa CI: €24,483 to 29,947) vs €22,503 (95% BCa CI: €20,328 to €24,944), *P* = 0.017, mean difference: −4504 (95% BCa CI: −7977 to −964)]. The sensitivity analysis excluding patients with cancer in the pancreatic head still showed higher total hospital costs for RPD compared with OPD [€29,172 (95% BCa CI: €26,162 to €32,408) vs €23,311 (95% BCa CI: €20,206 to €26,842), *P* = 0.019, mean difference: −5861 (95% BCa CI: −10,538 to −1,073)].

### Second Half of Robot-assisted Pancreatoduodenectomy Experience

**FIGURE. 1:**
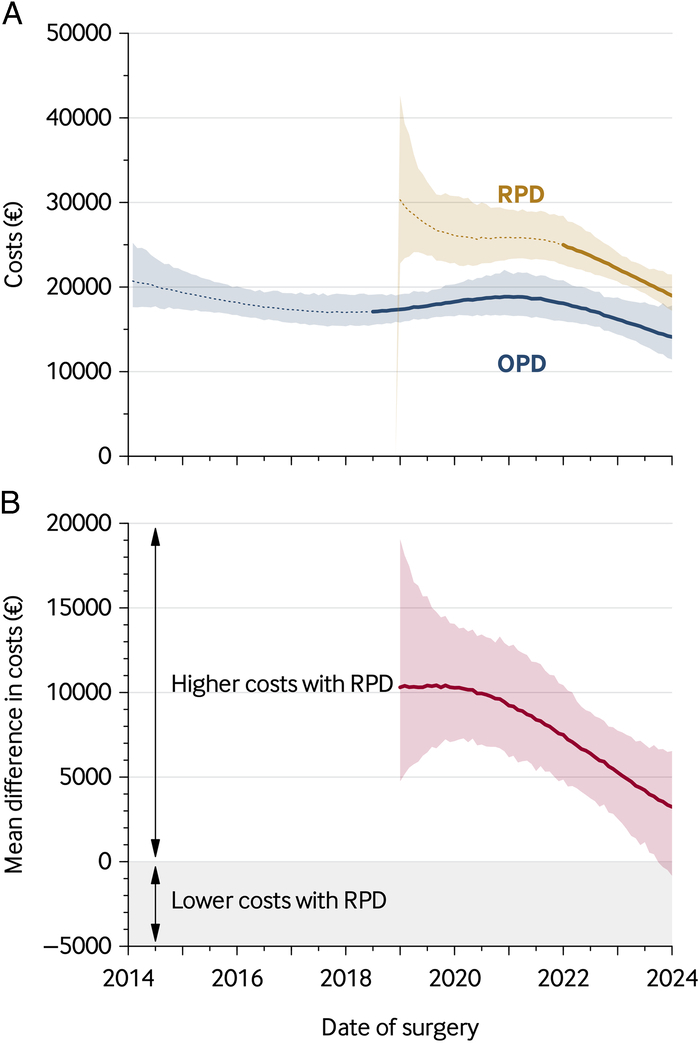
Total hospital costs of RPD versus OPD over time. A, Date of surgery (year) on *x*-axis and total hospital costs (euro) on *y*-axis for RPD (yellow) and OPD (blue) in the first (interrupted line) and second (continuous line) half of the experience. B, Date of surgery (year) on *x*-axis and relative difference in total hospital costs (euro) on *y*-axis between RPD and OPD.

The first half of the RPD experience (n = 107) was performed between February 2019 and December 2021, and the second half (n = 107) between January 2022 and June 2024. In the second half of the RPD experience [ie, excluding the first 50% (n = 107) RPD], total LOS decreased [12 days (7 to 23) to 9 (6 to 15), *P* < 0.001], as did length of primary stay [11 days (7 to 21) to 7 (5 to 11), *P* < 0.001], and more patients were discharged ≤6 days [19 (17.8%) to 35 (32.7%), *P* = 0.012]. Conversion rate was comparable between the first and second half of RPD patients (2.8% vs 3.7%, *P* = 0.701). Operative time decreased [373 minutes (341 to 411) to 310 (272 to 352), *P* < 0.001], as did the rates of POPF (48.6% to 31.8%, *P* = 0.012), DGE (33.6% to 15.0%, *P* = 0.001), bile leak [10 (9.3%) to 3 (2.8%), *P* = 0.045], PPH [16 (15.0%) to 6 (5.6%), *P* = 0.024], and organ failure [10 (9.3%) to 1 (0.9%), *P* < 0.001] in the second half of the RPD experience. Postoperative health care use decreased with fewer radiologic interventions [57 (53.3%) to 42 (39.3%), *P* = 0.040], reoperations [13 (12.1%) to 5 (4.7%), *P* = 0.049], MC/IC admissions [16 (15.0%) to 1 (0.9%), *P* < 0.001], and blood transfusions [19 (17.8%) to 8 (7.5%), *P* = 0.024]. Complication-related mortality was 0% (0/107 patients) in the second half. Consequently, the mean total hospital costs of RPD decreased by €11,058 in the second half (€32,836 to €21,778, *P* = 0.001). Both intraoperative costs (€12,749 to €11,741, *P* < 0.001) and postoperative costs (€20,087 to €10,037, *P* = 0.002) decreased (Table 3). Details on total hospital costs over time are presented in Figure 1.

**TABLE 3:** Outcome and Costs of Robot-assisted Pancreatoduodenectomy (First Versus Second Half)

	RPD first half (n = 107)	RPD second half (n = 107)	*P*	Mean difference (costs)
Postoperative complications				
POPF (grade B/C)	52 (48.6)	34 (31.8)	**0.012**	NA
DGE (grade B/C)	36 (33.6)	16 (15.0)	**0.001**	NA
Bile leak (grade B/C)	10 (9.3)	3 (2.8)	**0.045**	NA
PPH (grade B/C)	16 (15.0)	6 (5.6)	**0.024**	NA
Chyle leak (grade B/C)	0	0	NA	NA
Wound infection (CDC)			0.131	NA
Superficial	10 (9.3)	3 (2.8)	NA	NA
Deep	0	1 (0.9)	NA	NA
Intra-abdominal	12 (11.1)	17 (16.0)	NA	NA
Organ failure	10 (9.3)	1 (0.9)	**0.005**	NA
Postoperative health care use				
Length of primary stay (d)	11 (7 to 21)	7 (5 to 11)	**<0.001**	NA
Total length of stay (d)*	12 (7 to 23)	9 (6 to 15)	**<0.001**	NA
≤6 d	19 (17.8)	35 (32.7)	**0.012**	NA
Diagnostic imaging	78 (72.9)	66 (61.7)	0.080	NA
Endoscopy	47 (43.9)	38 (35.5)	0.209	NA
Radiologic intervention	57 (53.3)	42 (39.3)	**0.040**	NA
Reoperation	13 (12.1)	5 (4.7)	**0.049**	NA
MC/IC admission	16 (15.0)	1 (0.9)	**<0.001**	NA
Blood transfusion	19 (17.8)	8 (7.5)	**0.024**	NA
Readmission	25 (23.4)	24 (22.4)	0.871	NA
Outpatient hospital care				NA
ER visit	26 (24.3)	19 (17.8)	0.240	NA
Outpatient clinic	0	0	NA	NA
Mortality (in-hospital/30 d)	3 (2.8)	1 (0.9)	0.313	NA
Costs (EUR)				
Total intraoperative costs	**12,749 (12,574 to 12,943)**	**11,741 (11,526 to 11,979)**	**<0.001** **†**	**−1008 (−1314 to −700)**
Fixed	6440	6440	NA	NA
Additional	6309 (6134 to 6503)	5301 (5087 to 5539)	**<0.001** †	**−1008 (−1314 to −700)**
Total postoperative costs	**20,087 (15,992 to 24,864)**	**10,037 (8,498 to 11,714)**	**0.002** †	**−10,050 (−15,119 to −5681)**
Admission	15,502 (12,417 to 19,249)	7729 (6718 to 8819)	**0.005** †	**−7773 (−11,627 to −4501)**
Outpatient hospital care	88 (58 to 118)	50 (33 to 68)	NA‡	**−38 (−78 to 0)**
Diagnostic imaging	817 (644 to 1014)	428 (324 to 553)	**0.002** †	**−389 (−621 to −173)**
Endoscopy	722 (526 to 931)	526 (363 to 708)	0.163†	−196 (−465 to 66)
Radiologic interventions	1897 (1379 to 2499)	880 (609 to 1189)	**0.006** †	**−1017 (−1684 to −402)**
Surgical reinterventions	765 (388 to 1163)	310 (118 to 556)	0.074†	−455 (−950 to 17)
Other	296 (171 to 438)	113 (29 to 221)	NA‡	**−183 (−371 to 21)**
Total costs (intraoperative and postoperative)	**32,836 (28,789 to 37,554)**	**21,778 (20,209 to 23,476)**	**0.001** †	**−11,058 (−16,062 to −6671)**

Bold values are the total costs or showed a stastically significant difference between the groups.

Values are presented in medians with interquartile ranges and frequencies with percentages (%). Costs are reported as means with mean differences and their 95% BCa CIs.

* Total length of stay includes readmission(s).

† *P* value was generated based on mean difference between costs.

‡ No *P* value could be generated due to low number and variation in data.

CDC indicates Centers for Disease Control; EUR, euro; NA, not applicable.

### Second Half of Open Pancreatoduodenectomy Experience

The first half of the OPD experience (n = 187) was performed between January 2014 and June 2018, and the second half (n = 187) between June 2018 and December 2023. To mitigate any time bias, we excluded the first 50% of OPD procedures: total LOS after OPD did not differ [12 days (8 to 21) to 12 (7 to 22), *P* = 0.464], whereas the rates of POPF [40 (22.0%) to 65 (34.8%), *P* = 0.007] and chyle leak [4 (2.1%) to 12 (6.4%), *P* = 0.041] increased, and organ failure decreased [18 (9.7%) to 8 (4.3%), *P* = 0.039]. The rate of radiologic interventions and ER visits increased in the second half. Admission to MC/IC [30 (16.0%) to 14 (7.5%), *P* = 0.010] and in-hospital/30-day mortality [10 (5.3%) to 2 (1.1%), *P* = 0.019] decreased in the second half. Mean total hospital costs did not change significantly (€24,025 to €21,013, *P* = 0.210), see Supplemental File 3 (Supplemental Digital Content 1, http://links.lww.com/SLA/F407). Details on total hospital costs over time are presented in Figure 1.

### Clinical Outcome in the Second Half

When comparing the clinical outcomes after RPD (n = 107) and OPD (n = 187) both in the second half: total LOS was 3 days shorter after RPD [9 days (6 to 15) vs 12 (7 to 22), *P* < 0.001]. Length of primary stay was 4 days shorter after RPD [7 days (5 to 11) vs 11 (7 to 17), *P* < 0.001] and more patients were discharged ≤6 days after RPD [35 (32.7%) vs 28 (15.0%), *P* < 0.001], compared with OPD. Fewer patients were operated for cancer in the pancreatic head in the RPD group [26 (24.3%) vs 88 (47.1%), *P* < 0.001]. Operative time remained longer in the RPD group compared with OPD [310 minutes (272 to 352) vs 252 (221 to 305), *P* < 0.001] and intraoperative blood loss remained lower with RPD [150 mL (100 to 250) vs 300 (200 to 500), *P* < 0.001]. Also, PACU stay was shorter after RPD [6 hours (5 to 7) vs 7 (5 to 18), *P* < 0.001]. The rates of DGE [16 (15.0%) vs 54 (28.9%), *P* = 0.007], chyle leak [0 (0.0%) vs 12 (6.4%), *P* = 0.007], and wound infections [21 (19.7%) vs 74 (39.6%), *P* = 0.002] were lower after RPD, as compared with OPD. No differences were found in the other procedure-specific complications. Sensitivity analysis without cancer in the pancreatic head in the second half also showed no significant difference in the rate of POPF, with 35.8% after RPD versus 44.4% after OPD (*P* = 0.240). Fewer patients were admitted to the MC/IC after RPD [1 (0.9%) vs 14 (7.5%), *P* = 0.014]. No difference in in-hospital/30-day mortality was found between RPD and OPD [1 (0.9%) vs 2 (1.1%), *P* = 0.912]. For the second half, baseline characteristics are presented in Supplemental File 4 (Supplemental Digital Content 1, http://links.lww.com/SLA/F407) and intraoperative and postoperative outcomes in Table 4.

**TABLE 4:** Outcome and Costs of RPD Versus OPD (Second Half Only)

	RPD second half (n = 107)	OPD second half (n = 187)	*P*	Mean difference (costs)
Intraoperative outcomes				
Conversion	4 (3.7)	NA	NA	NA
Operative time (min)	310 (272 to 352)	252 (221 to 305)	**<0.001**	NA
Anesthesia time (min)	63 (57 to 72)	61 (51 to 71)	0.201	NA
Blood loss (mL)	150 (100 to 250)	300 (200 to 500)	**<0.001**	NA
Vein resection	4 (3.7)	15 (8.0)	0.151	NA
Arterial resection	0	1 (0.5)	0.449	NA
Small MPD	62 (58.5)	117 (68.0)	0.107	NA
Soft pancreatic texture	67 (63.8)	105 (62.5)	0.827	NA
PACU stay (h)	6 (5 to 7)	7 (5 to 18)	**<0.001**	NA
Postoperative complications				
POPF (grade B/C)	34 (31.8)	65 (34.8)	0.602	NA
DGE (grade B/C)	16 (15.0)	54 (28.9)	**0.007**	NA
Bile leak (grade B/C)	3 (2.8)	14 (7.5)	0.098	NA
PPH (grade B/C)	6 (5.6)	18 (9.6)	0.226	NA
Chyle leak (grade B/C)	0	12 (6.4)	**0.007**	NA
Wound infection (CDC)			**0.002**	NA
Superficial	3 (2.8)	16 (8.6)	NA	NA
Deep	1 (0.9)	0	NA	NA
Intra-abdominal	17 (16.0)	58 (31.0)	NA	NA
Organ failure	1 (0.9)	8 (4.3)	0.109	NA
Postoperative health care use				
Length of primary stay (d)	7 (5 to 11)	11 (7 to 17)	**<0.001**	NA
Total length of stay (d)*	9 (6 to 15)	12 (7 to 22)	**<0.001**	NA
≤6 d	35 (32.7)	28 (15.0)	**<0.001**	NA
Diagnostic imaging	66 (61.7)	134 (71.7)	0.078	NA
Endoscopy	38 (35.5)	70 (37.4)	0.743	NA
Radiologic intervention	42 (39.3)	86 (46.0)	0.262	NA
Reoperation	5 (4.7)	11 (5.9)	0.660	NA
MC/IC admission	1 (0.9)	14 (7.5)	**0.014**	NA
Blood transfusion	8 (7.5)	18 (9.7)	0.524	NA
Readmission	24 (22.4)	30 (16.0)	0.174	NA
Outpatient hospital care				NA
ER visit	19 (17.8)	32 (17.1)	0.888	NA
Outpatient clinic	0	2 (1.1)	0.283	NA
Mortality (in-hospital/30 d)	1 (0.9)	2 (1.1)	0.912	NA
Costs (EUR)				
Total intraoperative costs	**11,741 (11,519 to 11,987)**	**5543 (5439 to 5649)**	**<0.001** **†**	**−6197 (−6465 to −5948)**
Fixed	6440‡	1947‡	**<0.001** **†**	**−4493** **‡**
Additional	5301 (5080 to 5547)	3597 (3492 to 3703)	**<0.001** †	**−1704 (−1972 to −1455)**
Total postoperative costs	**10,037 (8468 to 11,748)**	**15,468 (13,124 to 18,113)**	**0.003** †	**5431 (2432 to 8614)**
Admission	7729 (6719 to 8755)	12,697 (10,890 to 14,799)	**<0.001** †	**4967 (2808 to 7408)**
Outpatient hospital care	50 (33 to 68)	49 (34 to 65)	0.913†	−1 (−29 to 24)
Diagnostic imaging	428 (323 to 546)	566 (469 to 675)	0.092†	138 (−27 to 303)
Endoscopy	526 (371 to 692)	498 (383 to 624)	0.790†	−28 (−241 to 191)
Radiologic interventions	880 (617 to 1166)	1247 (954 to 1595)	0.105†	367 (−84 to 836)
Surgical reinterventions	310 (118 to 556)	339 (185 to 494)	0.880†	29 (−330 to 342)
Other	113 (29 to 229)	146 (74 to 237)	0.690†	34 (−147 to190)
Total costs (intraoperative and postoperative)	**21,778 (20,206 to 23,502)**	**21,013 (18,661 to 23,662)**	**0.644** †	**−764 (−3843 to 2447)**
Excl robot purchase costs	19,628 (18,056 to 21,352)	21,013 (18,661 to 23,662)	0.395†	1386 (−1693 to 4597)

Bold values are the total costs or showed a stastically significant difference between the groups.

Values are presented in medians with interquartile ranges and frequencies with percentages (%). Costs are reported as means with mean differences and their 95% BCa CIs.

* Total length of stay includes readmission(s).

† *P* value was generated based on mean difference between costs.

‡ No CIs could be generated.

CDC indicates Centers for Disease Control; EUR, euro; MPD, main pancreatic duct; NA, not applicable.

### Hospital Costs in the Second Half

In the second half, no difference was found in mean total hospital costs between RPD and OPD (€21,778 vs €21,013, *P* = 0.644). Intraoperative costs remained higher after RPD compared with OPD (€11,741 vs €5543, *P* < 0.001), whereas postoperative costs were lower after RPD (€10,037 vs €15,468, *P* = 0.003; Table 4).

When excluding robot purchase costs, total hospital costs remained similar between RPD and OPD (€19,628 vs €21,013, *P* = 0.395). The recently proposed AMP finance structure (€1850 per robot procedure) did not have an impact on total hospital costs of RPD versus OPD in the second half [€21,478 (95% BCa CI: €19,863 to €23,234) vs €21,013 (95% BCa CI: €18,661 to €23,662), *P* = 0.760, mean difference: −464 (95% BCa CI: −3463 to 2645)]. Sensitivity analysis of the RPD mastery learning curve showed higher total hospital costs after RPD mastery (>84 RPDs) compared with the second half of OPD patients [€25,073 (95% BCa CI: €22,599 to €27,902) vs €21,013 (95% BCa CI: €18,669 to €23,601), *P* = 0.041, mean difference: −4060 (95% BCa CI: −8143 to −109)]. Sensitivity analysis excluding patients with cancer in the pancreatic head showed no difference in total hospital costs between RPD and OPD in the second half [€22,605 (95% BCa CI: €20,593 to €24,702) vs €21,063 (95% BCa CI: €18,286 to €24,202), *P* = 0.454, mean difference: −1542 (95% BCa CI: −5261 to 2397)].

## DISCUSSION

This largest cost analysis during the implementation of 214 RPDs showed that hospital stay was 2 days shorter after RPD and mean total hospital costs were higher for RPD compared with 374 patients undergoing OPD (€27,307 vs €22,503, *P* = 0.010). This cost difference disappeared in the second half of the experience as the clinical outcome of RPD improved, reflected in 3 days shorter hospital stay, 16.8% less POPF, 18.6% less DGE, 6.5% less bile leak, 9.4% less PPH, and 14.1% less MC/IC admission, as compared with the first half of RPD procedures. Consequently, the mean total hospital costs of RPD decreased by €11,058 per patient and became similar for RPD and OPD. In the second half of the experience, RPD, as compared with OPD, was associated with 3 days shorter hospital stay, 150 mL less blood loss, 13.9% less DGE, 6.4% less chyle leak, 19.9% less wound infections, and 6.6% less MC/IC admissions.

As this is the first large-scale cost analysis of RPD versus OPD which takes the impact of the learning curve into account, only the clinical patient outcome can be compared with earlier single-center cohorts. A comparative series in 99 patients undergoing RPD and 276 undergoing OPD from Heidelberg, Germany, reported more patients without a complication after RPD compared with OPD (50% vs 19%, *P* < 0.001), whereas the LOS (13 vs 12 days, *P* = 0.444) and POPF rate (21.1% vs 23.3%, *P* = 0.858) were similar between RPD and OPD.^24^ The current study showed fewer conversions as compared with the Heidelberg series (3.3% vs 17.8%, respectively). Also, the LOS (9 vs 13 days) and reoperation rate (8.4% vs 14.4%) after RPD were lower in the current study, whereas the POPF rate was higher (40.2% vs 21.1%). Improvements during a growing RPD experience were also reported in the largest (noncomparative) single-center Western series on 500 RPDs from Pittsburgh: reduced operative time, conversion rate, intraoperative blood loss, LOS (8 days in the total cohort to 6 days in the last 100 RPDs, *P* < 0.001), major complications, and POPF rate (7.8% to 3.0%, *P* = 0.002).^25^ The European consortium on Minimally Invasive Pancreatic Surgery registry reported on postoperative morbidity and mortality after 835 RPDs (including the learning curve) from 45 centers in 14 European countries. After RPD, the median LOS was 12 days with a POPF rate of 25.4%.^26^ The 1.9% mortality after RPD in the current study (0.9% in the second half) is comparable to 1.8% in the Pittsburgh series (3.0% in the last 100 RPD), and less than 6.0% in the Heidelberg study and 4.1% in the European consortium on Minimally Invasive Pancreatic Surgery registry (both including the learning curve).^24–26^

For the cost analysis, previous studies reported either higher costs for RPD or similar costs between approaches. A recent meta-analysis including 16 studies reported €13,149 higher costs of RPD compared with OPD (*P* = 0.013).^27^ A single-center study from Tampa, USA, reported higher direct costs in 205 RPDs compared with 181 OPDs (*P* = 0.040).^10^ A single-center study from Pisa, Italy, showed higher direct costs in 20 RPDs compared with 40 OPDs, whereas costs were similar when excluding robot purchase/maintenance costs (*P* = 0.076).^11^ In contrast, Aguayo et al^28^ (830 RPDs vs 75,996 OPDs) and Baker et al^29^ (22 RPDs vs 49 OPDs) showed no difference in total costs between approaches (*P* = 0.280 and *P* > 0.050, respectively). None of these studies specified the impact of the learning curve on RPD costs.

Interestingly, in the current study, the rates of several complications (ie, chyle leakage and wound infection) were reduced after RPD as compared with OPD. In addition, RPD was associated with a lower DGE rate in the second half. Although the rates of POPF and ER visits after RPD were higher in the complete cohort, these rates were comparable between RPD and OPD in the second half. It is important to note that the incidence of the low-risk-of-complication indication cancer in the pancreatic head was 22.9% in the RPD group compared with 48.7% OPD. Thus, more high-risk patients were selected for RPD. In the total cohort, the POPF rate was initially 11.7% higher after RPD compared with OPD (*P* = 0.004). After excluding cancer in the pancreatic head as an indication (n = 357), the risk difference of 8.7% was not significant (*P* = 0.093). In the second half, the POPF rate was comparable between groups (31.8% RPD vs 34.8% OPD, *P* = 0.602). After excluding cancer in the pancreatic head in the second half, fistula risk was nonsignificantly lower after RPD than OPD (35.8% RPD vs 44.4% OPD, *P* = 0.411). This finding is consistent with the results of the recently completed DIPLOMA-2 trial, which reported a lower POPF rate after RPD compared with OPD.^30^

Ultimately, large-scale randomized trials should confirm the benefits of RPD over OPD. Until now, three such studies have been performed.^6,7,31^ The single-center phase 2 EUROPA-trial from Heidelberg showed similar Comprehensive Complication Index rates between RPD and OPD, whereas the rate of major pancreas-specific complications was higher after RPD (58.6% vs 33.3%, *P* = 0.046) with no significant difference in POPF rate between groups (37.9% vs 21.2%, *P* = 0.148). This trial reported higher hospital costs (without purchase costs) after RPD compared with OPD (*P* = 0.011). However, the high RPD conversion rate (23%) implies that the learning curve was not yet completed at the time of the trial.^6^ The multicenter randomized trial of Liu and colleagues from China reported a 2.5-day shorter hospital stay after RPD compared with OPD (11 vs 13.5 days, *P* = 0.029) with similar rates of severe complications and mortality. Liu et al^7^ reported higher procedure costs of RPD compared with OPD, albeit without specification. The results of the international European DIPLOMA-2 trial (ISRCTN27483786) have yet to be published.^31^ Notably, randomized trials cannot provide sufficient granular data on costs and improvements during an entire single-center learning curve, as presented in our current study, as the number of patients included per center in a randomized trial is typically small.

It is important to note that our center completed the LAELAPS-3 training program before starting with RPD independently and that the first RPD was performed after 55 laparoscopic pancreatoduodenectomies which may have accelerated the RPD learning curve.^5^ Improvement of patient outcomes after the learning curve subsequently translated into lower hospital costs. Our group previously reported that median hospital costs of OPD ranged from €17,482 for patients without complications to €55,623 for patients with PPH.^32^ Pancreatic ductal adenocarcinoma, severity of complication, and postoperative infections were also predictors for increased costs.^32^ A relation between postoperative complications and hospital costs was also seen in the present cohort. In the complete cohort, higher postoperative complications after RPD compared with OPD caused increased postoperative health care use and subsequent higher total hospital costs, also in the sensitivity analysis without cancer in the pancreatic head. In the second half, the lower postoperative costs outweigh the higher intraoperative costs for RPD, with the total cost of RPD resembling the costs of OPD. Other adjustments in patient management may also have had an impact on postoperative outcomes and hospital costs, such as the implementation of the ERAS protocol and intraoperative techniques.^20^

Sensitivity analysis for the mastery cutoff (>84 RPDs) still showed €4060 higher total hospital costs after completion of the mastery learning curve than the second half of OPD patients (*P* = 0.041). The break-even point for total hospital costs of RPD and OPD apparently lies beyond the mastery learning curve and even further in the second half of the robotic experience as shown in Figure 1. The recently proposed AMP finance structure (€1850 per robot procedure) did not impact the total hospital costs of RPD versus OPD. Hence, in case of subtle variation in costs per procedure between countries and centers, the results of the present study can probably be translated into other countries/practices.

The results of the present study should be interpreted considering several limitations. First, this is a single-center, post hoc analysis from a high-volume center, which limits the generalizability of the results. However, an economic break-even point between 84 and 107 RPDs suggests that RPD is probably best implemented in high-volume centers rather than small centers to promptly achieve economic justification. The Miami^2^ and Brescia^3^ guidelines recommend a minimum of 20 minimally invasive pancreatoduodenectomy procedures per center per year. The current study supports this advice as even with this volume it will require 5 years before RPD becomes a cost-effective procedure as compared with OPD. Second, the study used a retrospective design. Although most data were collected in the prospectively maintained nationwide Dutch Pancreatic Cancer Audit, no differentiation could be made between pancreatic ductal adenocarcinoma and distal cholangiocarcinoma in the sensitivity analyses. Third, selection bias may have occurred, but the RPD selection criteria were applied to the OPD patients to minimize selection bias. Fourth, developments over time (eg, ERAS protocol and PORSCH trial^20,33^) may have influenced clinical and economic outcomes. Comparable outcomes were found between the first and second half of OPD patients. Therefore, to minimize time bias, the second half of RPD patients was compared with the second half of OPD patients (instead of all OPDs). A similar inclusion period for RPD and OPD would have resulted in an OPD cohort that was considerably smaller (n = 61) due to the 2:1 randomization (RPD:OPD) in the DIPLOMA-2 trial during this time frame. Fifth, the purchase and maintenance costs of the robotic system and the costs of complications may differ per country, which may affect the generalizability of the findings. To enable application in other practices, we performed a sensitivity analysis without the robot purchase and maintenance costs. Sixth, this study did not include data on out-of-hospital care, work loss, and oncological outcomes, including time to start adjuvant chemotherapy. These factors may also be associated with considerable costs for wound care, health care use at home, and later return to work. These costs may have favored RPD. The latter should be investigated by future randomized studies, such as the ongoing DIPLOMA-2×2 trial (ISRCTN27483786). Seventh, there may be variability between surgeons in the present study cohort as 7 surgeons perform OPD and 4 surgeons RPD in our center, which could alter the results, although this does reflect real-life practice. The main strength of this study lies in the large sample size allowing for the analysis beyond the learning curve and the robust economic analysis.

## CONCLUSIONS

The present study showed that implementing RPD is associated with considerable additional costs when compared with OPD. In the second half of the RPD experience these differences disappeared, as outcomes improved and total RPD hospital costs decreased with over €11,000 per patient. Beyond the learning curve, RPD is associated with improved clinical outcomes compared with OPD, particularly in terms of LOS and complications. These findings suggest that with increasing surgical experience, RPD is a viable alternative to OPD in selected patients in high-volume centers. Surgeons and hospitals should invest in shortening the learning curve of these complex and costly procedures, for instance through dedicated integrated training programs. Future studies, especially large-scale randomized trials, are needed to confirm the present findings.

## Supplementary Material
