# Supplementary material for: Cost-analysis of Implementing Robot-assisted Versus Open Pancreatoduodenectomy
Source: Ann Surg. 2025 Feb 10;284(2):353–62. doi: 10.1097/SLA.0000000000006665 (PMC13344404; doi:10.1097/SLA.0000000000006665)
Supplement: Supplementary file 1 [file sla-284-353-s001.docx]

# Supplementary file 1: CHEERS 2022 checklist

| **Topic** | **No.** | **Item** | **Location where item is reported** |
| --- | --- | --- | --- |
| **Title** |  |  |  |
|  | 1 | Identify the study as an economic evaluation and specify the interventions being compared. | Title page |
| **Abstract** |  |  |  |
|  | 2 | Provide a structured summary that highlights context, key methods, results, and alternative analyses. | Abstract |
| **Introduction** |  |  |  |
| **Background and objectives** | 3 | Give the context for the study, the study question, and its practical relevance for decision making in policy or practice. | Introduction, last paragraph |
| **Methods** |  |  |  |
| **Health economic analysis plan** | 4 | Indicate whether a health economic analysis plan was developed and where available. | Not reported |
| **Study population** | 5 | Describe characteristics of the study population (such as age range, demographics, socioeconomic, or clinical characteristics). | Methods, Patient selection |
| **Setting and location** | 6 | Provide relevant contextual information that may influence findings. | Methods, Patient selection & Data collection |
| **Comparators** | 7 | Describe the interventions or strategies being compared and why chosen. | Methods, Patient selection & Local standard of care |
| **Perspective** | 8 | State the perspective(s) adopted by the study and why chosen. | Methods, Data collection |
| **Time horizon** | 9 | State the time horizon for the study and why appropriate. | Methods, Patient selection & Data collection |
| **Discount rate** | 10 | Report the discount rate(s) and reason chosen. | Methods, Costs |
| **Selection of outcomes** | 11 | Describe what outcomes were used as the measure(s) of benefit(s) and harm(s). | Methods, Primary outcomes, Secondary outcomes, Costs |
| **Measurement of outcomes** | 12 | Describe how outcomes used to capture benefit(s) and harm(s) were measured. | Not reported |
| **Valuation of outcomes** | 13 | Describe the population and methods used to measure and value outcomes. | Not reported |
| **Measurement and valuation of resources and costs** | 14 | Describe how costs were valued. | Methods, Costs |
| **Currency, price date, and conversion** | 15 | Report the dates of the estimated resource quantities and unit costs, plus the currency and year of conversion. | Methods, Costs |
| **Rationale and description of model** | 16 | If modelling is used, describe in detail and why used. Report if the model is publicly available and where it can be accessed. | Not applicable |
| **Analytics and assumptions** | 17 | Describe any methods for analysing or statistically transforming data, any extrapolation methods, and approaches for validating any model used. | Not reported |
| **Characterising heterogeneity** | 18 | Describe any methods used for estimating how the results of the study vary for subgroups. | Not reported |
| **Characterising distributional effects** | 19 | Describe how impacts are distributed across different individuals or adjustments made to reflect priority populations. | Methods, Costs |
| **Characterising uncertainty** | 20 | Describe methods to characterise any sources of uncertainty in the analysis. | Methods, Statistical Analysis |
| **Approach to engagement with patients and others affected by the study** | 21 | Describe any approaches to engage patients or service recipients, the general public, communities, or stakeholders (such as clinicians or payers) in the design of the study. | Not reported |
| **Results** |  |  |  |
| **Study parameters** | 22 | Report all analytic inputs (such as values, ranges, references) including uncertainty or distributional assumptions. | Not applicable |
| **Summary of main results** | 23 | Report the mean values for the main categories of costs and outcomes of interest and summarise them in the most appropriate overall measure. | Tables 2-4 |
| **Effect of uncertainty** | 24 | Describe how uncertainty about analytic judgments, inputs, or projections affect findings. Report the effect of choice of discount rate and time horizon, if applicable. | Not reported |
| **Effect of engagement with patients and others affected by the study** | 25 | Report on any difference patient/service recipient, general public, community, or stakeholder involvement made to the approach or findings of the study | Not reported |
| **Discussion** |  |  |  |
| **Study findings, limitations, generalisability, and current knowledge** | 26 | Report key findings, limitations, ethical or equity considerations not captured, and how these could affect patients, policy, or practice. | Discussion, paragraph 8 |
| **Other relevant information** |  |  |  |
| **Source of funding** | 27 | Describe how the study was funded and any role of the funder in the identification, design, conduct, and reporting of the analysis | Disclosures |
| **Conflicts of interest** | 28 | Report authors conflicts of interest according to journal or International Committee of Medical Journal Editors requirements. | Conflicts of interest |

Legend supplementary file 1: Reference: Husereau D, Drummond M, Augustovski F, et al. Consolidated Health Economic Evaluation Reporting Standards 2022 (CHEERS 2022) Explanation and Elaboration: A Report of the ISPOR CHEERS II Good Practices Task Force. Value Health 2022;25. <doi:10.1016/j.jval.2021.10.008>

# Supplementary file 2: Unit costs

| **Resources** | ***Unit*** | ***Cost, (€, 2023)*** | ***Reference*** |
| --- | --- | --- | --- |
| **Admission** |  |  |  |
| General ward stay | Day | 668,47 | DCM |
| Intensive care unit stay | Day | 2830,63 | DCM |
| Outpatient hospital care |  |  |  |
| Outpatient clinic visit | Visit | 124,56 | DCM |
| Emergency department visit | Visit | 267,80 | DCM |
| **Diagnostic imaging** |  |  |  |
| CT abdomen | Test | 269,85 | CL |
| CT thorax | Test | 269,85 | CL |
| CT brain | Test | 269,85 | CL |
| Abdominal ultrasound | Test | 190,94 | CL |
| X-thorax | Test | 85,29 | DCM |
| X-abdomen | Test | 93,38 | CL |
| PET CT total body | Test | 629,12 | CL |
| Echocardiogram | Test | 350,76 | CL |
| MRI abdomen | Test | 513,06 | CL |
| **Endoscopy** |  |  |  |
| Endoscopic nasogastric feeding tube placement | Procedure | 961,34 | CL |
| Cortrak feeding tube placement | Procedure | 480,67 | CL |
| Colonoscopy | Procedure | 968,41 | CL |
| Endoscopic ultrasound | Procedure | 847,03 | CL |
| Endoscopic sclerotherapy | Procedure | 1222,31 | CL |
| Bronchoscopy | Procedure | 962,50 | CL |
| Percutaneous endoscopic gastrostomy | Procedure | 1443,96 | CL |
| Video Assisted Thoracoscopic Surgery, drainage | Procedure | 3267,91 | CL |
| Gastroscopy | Procedure | 723,43 | CL |
| **Radiological interventions** |  |  |  |
| X-ray guided PTC drainage | Procedure | 1049,34 | CL |
| Ultrasound-guided PTC drainage | Procedure | 1200,46 | CL |
| Vascular stent placement | Procedure | 2119,47 | CL |
| Vascular embolization | Procedure | 2119,47 | CL |
| Diagnostic angiography | Procedure | 187,71 | CL |
| Central venous catheter placement | Procedure | 750,57 | CL |
| CT guided abdominal collection drainage | Procedure | 883,35 | CL |
| Cardiac catheterization | Procedure | 1758,92 | CL |
| Ultrasound guided abdominal collection drainage | Procedure | 1200,46 | CL |
| **Surgical procedures** |  |  |  |
| Relaparoscopy for complication | Procedure | 6857,17 | CL |
| Relaparotomy for complication | Procedure | 5768,57 | CL |
| **Intraoperative costs** |  |  |  |
| Cost operating room (no surgeons, housing, equipment, personnel, overhead) | Minute | 8,18 | ORC |
| Cost operating room (one surgeon, housing, equipment, personnel, overhead) | Minute | 11,52 | ORC |
| Cost operating room (two surgeons, housing, equipment, personnel, overhead) | Minute | 14,86 | ORC |
| Surgical set, open (or conversion) | Set | 1,946.56 | ORC |
| Surgical set, laparoscopic | Set | 1,776.91 | ORC |
| Surgical set, robot | Set | 4,289.60 | ORC |
| Amortized costs robot | Usage cost | 2150,00 | CL |
| **Other** |  |  |  |
| Blood transfusion, erythrocytes | Bag | 446,20 | CL |

Legend supplementary file 2: Costs are derived from the Dutch costing manual (DCM) [24], AMC cost ledger (2018) and ODV cost ledger (2023) (CL). Operating room/personnel costs are based on the publication from Patel et al (ORC)(2022). All costs are indexed to 2023.

# Supplementary file 3: Outcome and costs of open pancreatoduodenectomy (first vs second half)

|  | **OPD first half (n=187)** | **OPD second half (n=187)** | **P-value** | **Mean difference (costs)** |
| --- | --- | --- | --- | --- |
| **Postoperative complications** |  |  |  |  |
| POPF (grade B/C) | 40 (22.0%) | 65 (34.8%) | **0.007** | N/A |
| DGE (grade B/C) | 49 (26.2%) | 54 (28.9%) | 0.563 | N/A |
| Bile leak (grade B/C) | 17 (9.1%) | 14 (7.5%) | 0.574 | N/A |
| PPH (grade B/C) | 21 (11.2%) | 18 (9.6%) | 0.612 | N/A |
| Chyle leak (grade B/C) | 4 (2.1%) | 12 (6.4%) | **0.041** | N/A |
| Wound infection (CDC)  Superficial  Deep  Intraabdominal | 22 (11.8%)  3 (1.6%)  50 (26.7%) | 16 (8.6%)  0 (0.0%)  58 (31.0%) | 0.208 | N/A |
| Organ failure | 18 (9.7%) | 8 (4.3%) | **0.039** | N/A |
| **Postoperative healthcare use** |  |  |  |  |
| Length of primary stay (days) | 11 [8–20] | 11 [7–17] | 0.062 | N/A |
| Total length of stay (days)#  ≤6 days | 12 [8–21]  18 (9.6%) | 12 [7–22]  28 (15.0%) | 0.464  0.115 | N/A |
| Diagnostic imaging | 129 (69.0%) | 134 (71.7%) | 0.571 | N/A |
| Endoscopy | 64 (34.2%) | 70 (37.4%) | 0.518 | N/A |
| Radiologic intervention | 62 (33.2%) | 86 (46.0%) | **0.011** | N/A |
| Reoperation | 21 (11.2%) | 11 (5.9%) | 0.065 | N/A |
| MC/IC admission | 30 (16.0%) | 14 (7.5%) | **0.010** | N/A |
| Blood transfusion | 13 (7.1%) | 18 (9.7%) | 0.381 | N/A |
| Readmission | 24 (12.8%) | 30 (16.0%) | 0.377 | N/A |
| Outpatient hospital care  ER visit  Outpatient clinic | 13 (7.0%)  1 (0.5%) | 32 (17.1%)  2 (1.1%) | **0.003**  0.562 | N/A |
| Mortality (in-hospital/30-day) | 10 (5.3%) | 2 (1.1%) | **0.019** | N/A |
| **Costs (EUR)** |  |  |  |  |
| **Total intraoperative costs** | **5,688 (5,552**–**5,825)** | **5,543 (5,436**–**5,650)** | **0.110*** | -145 (-326 to 26) |
| Fixed | 1,947 | 1,947 | N/A | N/A |
| Additional | 3,742 (3,605–3,878) | 3,597 (3,489–3,704) | 0.110* | -145 (-326 to 26) |
| **Total postoperative costs** | **18,368 (14,918–22,340)** | **15,468 (13,137–18,084)** | **0.226*** | **-2,899 (-7,710 to 1,516)** |
| Admission | 15,284 (12,432–18,726) | 12,697 (10,947–14,760) | 0.192* | -2,587 (-6,591 to 1,044) |
| Outpatient hospital care | 21 (11–33) | 49 (34–64) | **0.005*** | **27 (8 to 46)** |
| Diagnostic imaging | 565 (459–685) | 566 (472–674) | 0.986* | 1 (-152 to 157) |
| Endoscopy | 600 (450–766) | 498 (384–623) | 0.324* | -102 (-305 to 94) |
| Radiologic interventions | 1,045 (769–1,356) | 1,247 (952–1,597) | 0.367* | 202 (-236 to 654) |
| Surgical reinterventions | 715 (438–999) | 339 (185–494) | ^ | -376 (-721 to -42) |
| Other | 78 (37–125) | 146 (70–240) | 0.229 | 68 (-31 to 179) |
| **Total costs (intra- and postoperative)** | **24,025 (20,593–27,975)** | **21,013 (18,647–23,642)** | **0.210*** | **-3,012 (-7,828 to 1,408)** |

Legend supplementary file 3: Values are presented in medians with interquartile ranges [IQR] and frequencies with percentages (%). Costs are reported as means with mean differences and their bias-corrected and accelerated 95% confidence intervals (95% BCa CI). OPD, open pancreatoduodenectomy; POPF, postoperative pancreatic fistula; DGE, delayed gastric emptying; PPH, post-pancreatectomy hemorrhage; CDC, Centers for Disease Control; LOS, length of hospital stay; MC, medium care; IC, intensive care; ER, emergency room; EUR, euro. #Total length of stay includes readmission(s). *P-value was generated based on mean difference between costs. ^No p-value could be generated due to low number and variation in data.

# Supplementary file 4: Baseline characteristics of robot-assisted vs open pancreatoduodenectomy (second half only)

|  | **Total second half**  **(n=294)** | **RPD second half**  **(n=107)** | **OPD second half**  **(n=187)** | **P-value**** |
| --- | --- | --- | --- | --- |
| **Baseline characteristics** |  |  |  |  |
| Age (years) | 70 [62-76] | 71 [62–76] | 69 [62–76] | 0.692 |
| Female | 126 (42.9%) | 43 (40.2%) | 83 (44.4%) | 0.484 |
| BMI (kg/m2) | 24.9 [22.5-28.1] | 25.3 [22.4–28.7] | 24.6 [22.5–27.4] | 0.316 |
| ASA score 3 or higher | 80 (27.2%) | 23 (21.5%) | 57 (30.5%) | 0.096 |
| Cancer in the pancreatic head^#^ | 114 (38.8%) | 26 (24.3%) | 88 (47.1%) | **<0.001** |
| Tumor diameter (mm)* | 23 [17-33] | 25 [17–34] | 22 [17–30] | 0.297 |
| Neoadjuvant therapy  Chemoradiation  Chemotherapy  Radiotherapy | 5 (1.7%)  13 (4.4%)  1 (0.3%) | 1 (0.9%)  5 (4.7%)  1 (0.9%) | 4 (2.1%)  8 (4.3%)  0 (0.0%) | 0.503 |

Legend supplementary file 4: Values are presented in medians with interquartile ranges (IQR) and frequencies with percentages (%). RPD, robot-assisted pancreatoduodenectomy; OPD, open pancreatoduodenectomy; BMI, body-mass index; ASA, American Society of Anesthesiologists Physical Status Classification System. *Reported in 454/588 patients. **P-value was generated based on difference between RPD vs OPD cohort. ^#^Here defined as both pancreatic ductal adenocarcinoma and distal cholangiocarcinoma.
